# Multimodal ultrasound imaging of persistent urogenital sinus with uterus didelphys and double vagina malformation: A case report

**DOI:** 10.1097/MD.0000000000028477

**Published:** 2021-12-30

**Authors:** Yue Wang, Shourong Hu, Huifang Wang

**Affiliations:** a Department of Ultrasound, Peking University Shenzhen Hospital, Shenzhen, China; b Department of Ultrasound, Shenzhen Luohu People's Hospital, Third Affiliated Hospital of Shenzhen University, Shenzhen, China.

**Keywords:** bi-plane high-frequency ultrasound, double vagina, multimodal ultrasound, persistent urogenital sinus, uterus didelphys

## Abstract

**Rationale:**

Persistent urogenital sinus (PUG) with uterus didelphys and double vagina is a rare urogenital anomaly. The diagnosis is based on magnetic resonance examination and cystoscopy. To the best of our knowledge, there is no literature report of PUG diagnosed by ultrasound alone.

**Patient concern:**

A 23-year-old woman presented with atypical menstruation and recurrent hematuria for 13 years and recurrent lower abdominal pain for 12 years.

**Diagnosis:**

PUG was diagnosed through multiple ultrasound modalities, including transabdominal 2-dimensional ultrasound, transrectal bi-plane high-frequency ultrasound, and contrast-enhanced ultrasound. We diagnosed this malformation preoperatively by accurately measuring the length of urethra and common channel through multimodal ultrasound imaging.

**Interventions:**

Urethra separation and reconstruction, vaginal pull-through and artificial vaginoplasty, and bilateral hysterosalpingectomy were performed.

**Outcomes:**

The postoperative course was uneventful. She was urinating normally after half a year and used continuous vaginal dilatation to avoid stenosis.

**Lessons:**

PUG associated with uterus didelphys and double vagina is an extremely rare malformation of the reproductive system. Multimodal ultrasound imaging can be used to diagnose this malformation preoperatively clearly and to accurately measure the length of urethra and common channel, providing an imaging basis for preparing an operative plan.

## 1 Introduction

Persistent urogenital sinus (PUG) with uterus didelphys and double vagina is a rare urogenital anomaly.^[1]^ Understanding this condition is essential for making an accurate preoperative evaluation and thus choosing an appropriate surgical means. Previously reported that PUG had only been diagnosed through magnetic resonance imaging (MRI) and cystoscopy.^[2]^ Here, we report a PUG case with uterus didelphys and double vagina diagnosed through multiple ultrasound modalities, including transabdominal 2-dimensional ultrasound, transrectal bi-plane high-frequency ultrasound, and contrast-enhanced ultrasound (CEUS).

## 2 Case report

A 23-year-old Chinese woman was referred to our hospital following atypical menstruation and recurrent hematuria for 13 years, as well as recurrent lower abdominal pain for 12 years. She had undergone anoplasty due to congenital anal stenosis. Gynecological examination indicated a single orifice on the urogenital region where the urethra would be expected. There was a recess in the vaginal vestibule with an indentation of about 2 cm, while the rectal mucosa was everted in the anus. The karyotype was normal (46, XX).

**Figure 1:**
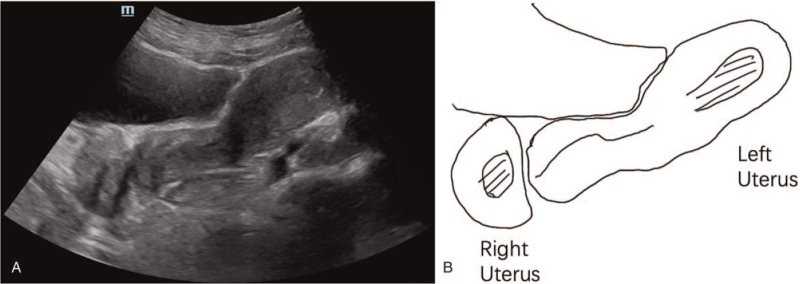
Two-dimensional transabdominal ultrasonography showing the longitudinal section of the left uterus and the transverse section of the right cervix.

**Figure 2:**
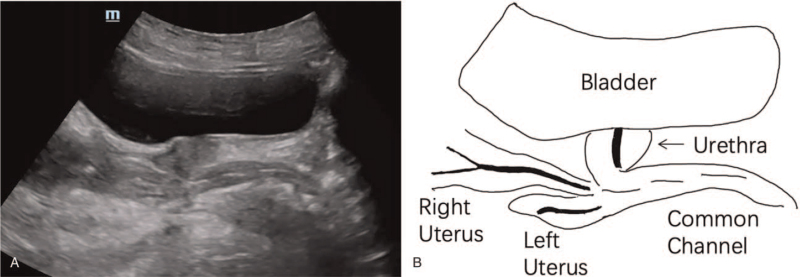
Two-dimensional transabdominal ultrasonography showing 2 vaginas and urethra.

**Figure 3:**
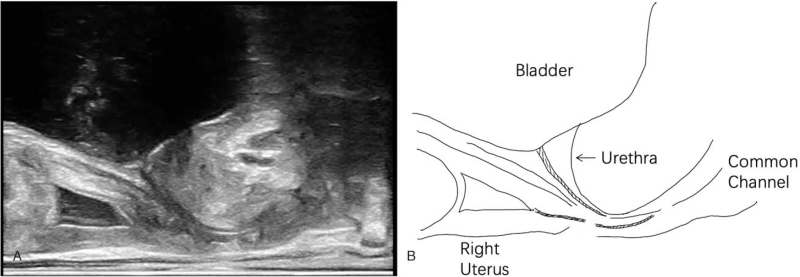
Transrectal biplanar high-frequency ultrasonography showing the urethra was bent at 90°, and the distal vagina was not identified.

Transabdominal two-dimensional ultrasonography and transrectal bi-plane high-frequency ultrasonography (Nuewa R9; Mindray Ultrasound, Shenzhen, China) found a double uterus with a double vagina. Distal end of both vaginas were not identified. The urethra was bent at 90° (Figs. 1–3). Bilateral ovarian cysts and bilateral tubal effusion were seen. The left kidney was smaller than the right, and there were no significant abnormalities in the bladder.

**Figure 4:**
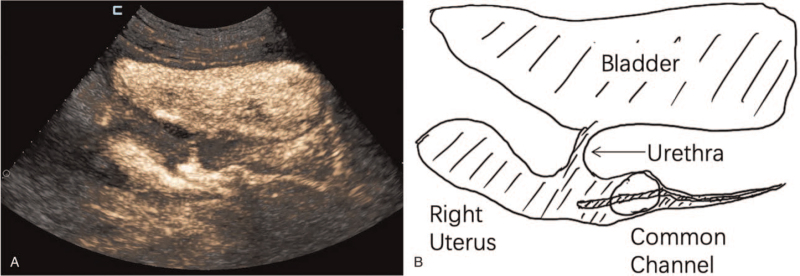
Contrast-enhanced 2-dimensional transabdominal ultrasonography showing the enhancement of the urethra, bladder, and vagina.

**Figure 5:**
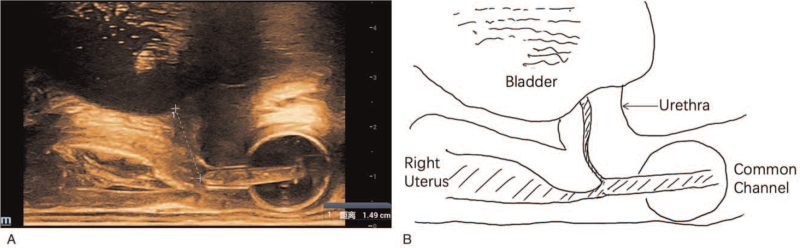
Contrast-enhanced 2-dimensional transrectal biplanar high-frequency ultrasonography showing the confluence of urethra and vagina.

A CEUS examination was performed on the next day. A No. 12 Foley catheter was inserted through the single orifice on the urogenital region, and 2.5 mL of normal saline was injected into the outer cavity tube to fix the catheter. SonoVue contrast agent was injected through the catheter (diluted with 1:10), while transabdominal ultrasound scan showed enhancement of 2 vaginas, urethra, and bladder (Fig. 4) (see Video, Video S1, Supplemental Digital Content, demonstrating the contrast agent passing through the common channel into the urethra, bladder, and right vagina by 2-dimensional transabdominal ultrasonography). A transrectal bi-plane high-frequency ultrasound scan demonstrated the 2 vaginas and the urethra merged into a common channel about 4.7 cm long and the urethra about 1.5 cm long (Fig. 5). The final diagnosis made by ultrasound was persistent urinary genital sinus (high confluence type) with uterus didelphys and double vagina malformation.

**Figure 6:**
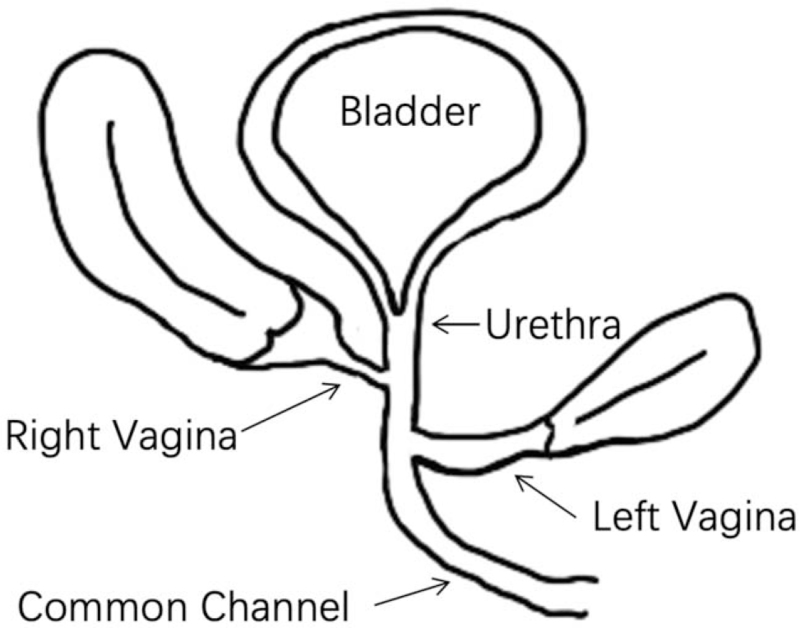
Sketch map to illustrate persistent urinary genital sinus with uterus didelphys and double vagina malformation.

**Figure 7:**
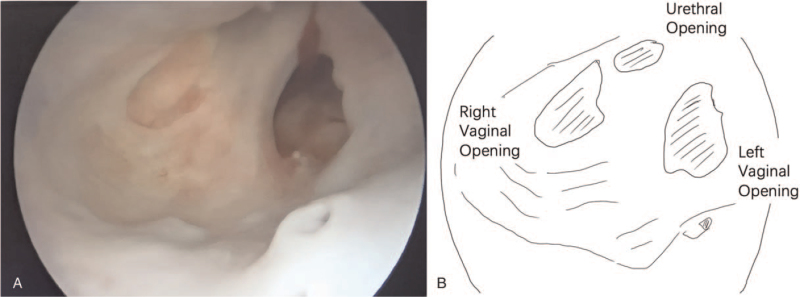
Urethroscopy showing the confluence of the urethra, right vagina, and left vagina.

The ureteroscopy and genitoscopy showed persistent urogenital sinus with an incomplete urethra and a septate vagina. The left vaginal opening was located at the left posterior and lower part of the right vaginal opening at the confluence. The urethra opening was located at the left anterior and upper part of the right vaginal opening (Figs. 6 and 7) (see Video, Video S2, Supplemental Digital Content, demonstrating the urethra and bilateral vagina's confluence urethroscopy). Genitography identified an incomplete longitudinal septum between the urethra and the 2 hemivaginas. The patient was submitted to surgical intervention. Laparoscopy showed 2 uteruses with the right fallopian tube without an umbrella end and remnants of the left fallopian tube. Urethra separation and reconstruction, vaginal pull-through and artificial vaginoplasty, and bilateral hysterosalpingectomy were successfully performed. The postoperative course was uneventful. She was urinating normally after half a year and used continuous vaginal dilatation to avoid stenosis.

## 3 Discussion

In the present case, there was an association of persistent urogenital sinus with a malformation of the Müllerian system (septate vagina and uterus didelphys). Conjugation of such anomalies has been reported 2 cases. One baby was submitted at 1 month of life for surgical intervention. Clitoroplasty, urethra separation and reconstruction, vaginal pull-through, and feminization plasty were successfully performed. The infant had normal development after 19 months of follow-up.^[3]^ But the other neonate died on postnatal day 1 and no surgical intervention was performed.^[4]^

Persistent urogenital sinus is a rare form of urogenital anomaly, with an incidence of approximately 6 in 100,000.^[1]^ The pathogenesis of PUG is the arrest of normal embryogenesis of the Mullerian tube and the failure of urethrovaginal separation, resulting in one orifice for the bladder and vagina to drain through. PUG is generally divided into 2 types based on the confluence of the urethra and vagina. A long urogenital sinus with a short vagina and a high urethral opening occurs when the defect occurs early in embryonic development. A short urogenital sinus with an almost normal length vaginal vestibule and low urethral opening results when the defect occurs later.^[5]^ In this case, the length of the urethra was 1.5 cm, and the length of the common channel was 4.7 cm, which was consistent with the high confluence type of urogenital sinus malformation.

PUG may exist alone or be associated with other congenital malformations in the urogenital tract, gastrointestinal tract, and cardiovascular system.^[6]^ It can also be associated with syndromes, including MC-Kusick-Kaufman syndrome, Bardet-Biedel syndrome, and hand-foot-genital syndrome.^[7]^ PUG was associated with uterus didelphys and double vaginal malformation in this case. The urethra and 2 vaginas formed a common channel. Previous authors have reported 2 cases of prenatal MRI diagnosis of PUG with duplicated hydrometrocolpos, including 1 with a vaginal septum.[^3^,^4^] PUG should be identified with vaginal atresia associated with a vaginal urethral fistula.

The relationship of the vagina to the bladder neck is the most critical factor for determining the type of vaginoplasty to be performed. For patients who have a short urethral length (<1.5 cm), postoperative urinary incontinence may occur if the preoperative evaluation is insufficient or an incorrect surgical method is selected.^[8]^ Therefore, preoperative imaging is crucial for the accurate anatomical evaluation of PUG. The diagnosis tended to be based on MRI examination and cystoscopy^[2]^ in PUG reports. Since the confluence of the urethra and vagina in PUG cannot be determined, and the length of the urethra and common channel cannot be accurately measured, conventional ultrasound should be combined with MRI and cystoscopy to diagnose PUG. In this case, various ultrasound imaging methods were used to accurately evaluate the confluence of the urethra and 2 vaginas before surgery and accurately measure the length of the urethra and common channel. The transrectal/transvaginal high-frequency linear array ultrasound probe had never been used in evaluating the vagina before. Since the probe is high frequency and can be inserted through anus, the structure of the vagina and urethra and their position relationship can be displayed clearly. CEUS can be used to observe the confluence of vaginas and urethra, accurately measure the length of urethra and common channel, and provide a comprehensive understanding of the anatomical abnormalities of PUG by dynamically observing the enhancement of the contrast agent. To the best of our knowledge, there is no literature report of PUG diagnosed by ultrasound alone.

In conclusion, PUG associated with uterus didelphys and double vagina is an extremely rare malformation of the reproductive system. Multimodal ultrasound imaging can be used to diagnose this malformation preoperatively clearly and to accurately measure the length of urethra and common channel, providing an imaging basis for preparing an operative plan.

## Author contributions

**Conceptualization:** Yue Wang, Huifang Wang.

**Data curation:** Shourong Hu.

**Formal analysis:** Yue Wang, Huifang Wang.

**Investigation:** Shourong Hu.

**Methodology:** Yue Wang, Shourong Hu.

**Project administration:** Huifang Wang.

**Supervision:** Huifang Wang.

**Validation:** Huifang Wang.

**Writing – original draft:** Yue Wang.

**Writing – review & editing:** Yue Wang.

## Supplementary Material

Supplemental Digital Content

## Supplementary Material

Supplemental Digital Content
